# Association of preoperative anemia and increased risk of blood transfusion and length of hospital stay in adults undergoing hip and knee arthroplasty: An observational study in a single tertiary center

**DOI:** 10.1002/hsr2.448

**Published:** 2021-12-10

**Authors:** Gustavo de Carvalho Duarte, Aguinaldo Pereira Catanoce, José Luiz Zabeu, Glaciano Nogueira Ribeiro, Mariangela Moschen, Nathália Aparecida Gonçalves de Oliveira, Dante Mario Langhi, José Francisco Comenalli Marques Júnior, Alfredo Mendrone‐Junior

**Affiliations:** ^1^ HHemo/Centro de Hemoterapia Celular em Medicina Campinas Brazil; ^2^ Department of Haematology Vera Cruz Hospital Campinas Brazil; ^3^ Fundação Pro Sangue Hemocentro de São Paulo São Paulo Brazil

**Keywords:** evidence‐based practice, orthopedic surgery, patient blood management, perioperative transfusion, preoperative anemia

## Abstract

**Background and aims:**

Anemia is a common feature in patients presenting for major elective surgery, and it is considered an independent risk factor associated with adverse outcomes. Although several studies suggest that preoperative anemia is associated with poor outcomes after elective orthopedic surgery, data are still scarce in middle‐ and low‐income countries where this problem may be even greater. The objective of this study was to evaluate the impact of preoperative anemia in clinical outcomes in patients submitted to hip and knee arthroplasty in a single tertiary hospital.

**Methods:**

Medical records of 234 adult patients submitted to knee and hip arthroplasty from January 2018 to July 2020 were retrospectively reviewed. Patient's demographics (ie, age and gender), pre‐ and postoperative hemoglobin level (Hb), allogeneic red blood cell (RBC) transfusion, intensive care admission, length of hospital stay, hospital readmissions, and mortality up to 30 days after the surgery were analyzed. Outcomes were evaluated according to preoperative anemia status based on World Health Organization (WHO) criteria.

**Results:**

Mean age was 70 years with a slight female predominance (57.6%). The prevalence of anemia was 30.7% (72/234) being more prevalent in female (33.3% vs 26.7%). Preoperative anemia was significantly associated with greater rate of blood transfusion (34.5% vs 5.6%; *P* = .001), prolonged length of hospital stay (6.48 days vs 3.36 days; *P* = .001), and higher rate of intensive care unit admission (47.2% and 29.6% *P* = .009). Preoperative anemia had no effect on 30‐day readmission and mortality for both males and females.

**Conclusions:**

Our retrospective study demonstrated that preoperative anemia is a common problem among orthopedic patients and is associated with increased transfusion risk and worse outcomes. Strategies to identify and treat anemic patients before elective surgery are critical to improving clinical outcomes.

## 1 INTRODUCTION

Anemia is a common feature in patients presenting for major elective orthopedic surgery, such as hip and knee arthroplasty, with a prevalence varying from 15% to 48%.^1^, ^2^ Previous reports support that preoperative anemia is an independent risk factor associated with worse postoperative outcomes, including length of stay, need for critical care admission, postoperative mortality, and blood transfusion. The anemia in this patient group may be secondary to blood losses, nutritional reasons, functional iron deficiency, or a combination of these, which may also increase the peri‐ and postoperative risk. A meta‐analysis with 24 observational studies (949 445 patients) compared outcomes after major surgery, including cardiac surgery, in patients with and without preoperative anemia. Patients with preoperative anemia were at an increased risk of major adverse outcomes including mortality, the odds ratio (OR) (95% CI) being 2.90 (2.30‐3.68), acute kidney injury, 3.75 (2.95‐4.76), and infection 1.93 (1.17‐3.18).^3^

Therefore, all patients for elective surgery in whom blood loss is expected to be >500 mL should have their hemoglobin checked preoperatively and be investigated if they are found to be anemic. Women are expected to be more prone to develop anemia than men, even presenting the same amount of blood loss during surgery, because they have lower circulating blood volumes and reduced red cell mass. This results in worse clinical outcomes with higher transfusions rates and hospital length of stay.^4^, ^5^ Rosencher et al, reported 1388 women who underwent surgery, showed that even nonanemic borderline hemoglobin (Hb 12.0‐12.9 g/dL) was associated with increased red cell transfusion and prolonged hospital stay compared to higher Hb levels (Hb ≥13 g/dL).^5^ Therefore, experts around the world advise that patients scheduled for a major surgical procedure and presenting with preoperative Hb less than 13 g/dL irrespective of gender should be considered at risk for adverse outcomes.^6^

Although studies in several health care settings have suggested that preoperative anemia is associated with poor outcomes after elective orthopedic surgery, data are scarce in middle and low‐incomes countries where this problem may be even greater.

## 2 OBJECTIVE

The objective of this study was to evaluate the impact of preoperative hemoglobin in clinical outcomes and transfusion risk in patients subjected to hip and knee arthroplasty in a single Brazilian tertiary hospital.

## 3 METHODS

Electronic medical records of all patients older than 18 years who underwent knee and hip arthroplasty from January 2018 to July 2020 were retrospectively reviewed (n = 434). Patients with no record of preoperative Hb were excluded (n = 200). The final cohort for analysis consisted of 234 patients.

Age, gender, pre‐ and postoperative Hb, number of RBC units transfused, intensive care admission during hospitalization, length of hospital stay (LOS) from the surgery day until hospital discharge, hospital readmissions, and mortality up to 30 days after the surgery were collected on paper case record. Preoperative Hb was considered the latest Hb analyzed within a maximum of 30 days before surgery. Patients were divided into preoperative anemic and nonanemic according to the WHO criteria.^7^ The primary outcome for this analysis was the allogeneic red blood cell (RBC) transfusion until 30 days after surgery. Secondary outcomes were LOS, intensive care admission during hospitalization, readmission, and mortality in the first 30 days.

A descriptive statistical analysis was performed, and continuous data were presented as mean ± SD (normally distributed). Categorical data were presented as the number and percentage of individuals in each category. The chi‐squared test was applied for proportions to compare proportions between the two groups. Independent samples *t*‐test and Mann–Whitney *U*‐test were used to compare continuous variables between the two independent groups, depending on the underlying distributions.

Data were analyzed using the NCSS 2020 Statistical Software (2020). NCSS, LLC. Kaysville, Utah, USA, ncss.com/software/ncss.

## 4 RESULTS

From the 234 patients included in the study, mean age was 70 (SD = 1.03) years with a slight female predominance (57.6%). Anemia was present in 30.7% of all patients (n = 72), 26.7% (n = 27), and 33.3% (n = 45) of males and females, respectively. Preoperative anemia was associated with a higher transfusion rate (34.7% vs 5.6%; *P* = .001), longer hospital stay (6.48 ± 1.2 vs 3.36 ± 0.3; *P* = .001), and higher rate of intensive care unit admission (47.2% vs 29.6%; *P* < .01) but not with higher number of RBC units transfused (2.76 ± 0.42 vs 3.0 ± 0.53; *P* = .76). Hospital readmissions and mortality during the first 30 days have no statistical difference between two groups (Table 1).

**TABLE 1:** Patient demographics and clinical outcomes

	All patients (n = 234)	Preoperative anemia (n = 72)	No preoperative anemia (n = 162)	Preoperative anemia × No preoperative anemia (*P* Value)
Age (Mean ± SD)	70.45 ± 1.03	74.06 ± 1.59	68.84 ± 1.29	<.05
Female	135 (57.6%)	45 (62.5%)	90 (55.6%)	‐
Male	99 (42.4%)	27 (37.5%)	72 (44.4%)	‐
Patients transfused	34 (14.5%)	25 (34.7%)	9 (5.6%)	.001
RBC units per patient transfused (Mean ± SD)	2.82 ± 0.33	2.76 ± 0.42	3.0 ± 0.53	.76
Preoperative hemoglobin (gr/dL) (Mean ± SD)	13.0 ± 0.11	10.97 ± 0.15	13.93 ± 0.08	.001
Postoperative hemoglobin (gr/dL) (Mean ± SD)	10.78 ± 0.13	9.54 ± 0.23	11.36 ± 012	.001
Length of stay (days) (Mean ± SD)	4.3 ± 0.4	6.48 ± 1.2	3.36 ± 0.3	.001
Intensive care admission	82 (35.0%)	34 (47.2%)	48 (29.6%)	<.01
30‐Day hospital readmission rate	25 (10.7%)	8 (11.1%)	17 (10.5%)	.9
30‐Day mortality	4 (1.7%)	2 (2.8%)	2 (1.2%)	.4

Abbreviations: gr/dL, grams per deciliter; SD, standard deviation.

The transfusion rate was higher in the previously anemic patients among men (22.2% vs 2.8%; *P* < .001) and in women (42.2% vs 7.8%; *P* < .01). The number of units of RBC transfused was not different in the anemic group, both in males (3.67 ± 1.12 vs 3.0 ± 3.0; *P* = .78) and females (2.47 ± 0.42 vs 3.0 ± 0.54; *P* = .501).

Preoperative anemia was also associated longer hospital length of stay regardless of gender, with 7.2 ± 2.4 days vs 3.4 ± 0.5 days for men (*P* < .05) and 6.0 ± 1.2 days vs 3.3 ± 0.3 days for women (*P* < .01). Among women, preoperative anemia was also related with postoperative intensive care admissions (53.3% vs 32.2%; *P* < .05).

There was no statistically significant difference in hospital readmissions rate and 30‐day mortality in preoperative anemic and nonanemic patients (Table 2).

**TABLE 2:** Clinical outcomes and red blood cell (RBC) transfusion according to gender and preoperative anemia status

	Males (n = 99)			Females (n = 135)		
Outcomes	Preoperative anemia (n = 27/26.7%)	No preoperative anemia (n = 72/72.3%)	*P* Value	Preoperative anemia (n = 45/33.3%)	No preoperative anemia (n = 90/66.6%)	*P* Value (95% confidence interval)
Preoperative hemoglobin (gr/dL) (Mean ± SD)	11.8 ± 0.22	14.5 ± 0.11	<.001	10.38 ± 0.16	13.48 ± 0.1	<.001
Postoperative hemoglobin (gr/dL) (Mean ± SD)	10.2 ± 0.7	11.9 ± 0.31	<.001	9.12 ± 0.54	10.96 ± 0.32	<.001
Patients transfused	6 (22.2%)	2 (2.8%)	<.01	19 (42.2%)	7 (7.8%)	<.001
RBC units transfused (mean ± SD)	0.81 ± 0.37	0.08 ± 0.07	<.01	1.04 ± 0.2	0.23 ± 0.1	<.001
RBC units per patient transfused (mean ± SD)	3.67 ± 1.12	3 ± 2	.78	2.47 ± 0.42	3 ± 0.54	.50
Length of hospital stay (mean ± SD)	7.2 ± 2.4	3.4 ± 0.5	<.05	6.0 ± 1.2	3.3 ± 0.3	<.01
Intensive care admission	10 (37.0%)	19 (26.4%)	.3	24 (53.3%)	29 (32.2%)	<.05
30‐Day hospital readmission	1 (3.7%)	6 (8.3%)	.4	7 (15.6%)	11 (12.2%)	.6
30‐Day mortality	1 (3.7%)	2 (2.8%)	.8	1 (2.2%)	0 (0%)	.2

Abbreviations: gr/dL, grams per deciliter; SD, standard deviation.

## 5 DISCUSSION

In this study, we evaluated the prevalence of anemia in patients admitted for elective hip and knee arthroplasty and its influence in postoperative clinical outcomes and in blood transfusion. Our findings are in consonance with the published literature showing a negative impact of anemia in the outcomes of individuals and transfusion risk.^5^, ^8^

The study showed an overall prevalence of preoperative anemia of 30.7% and an overall transfusion rate of 14.5% with 2.82 (± 0.33) units of RBC transfused per patient. As expected, anemia was more prevalent in female than male (33.3% vs 26.7%). Patients with anemia prior to the surgery were significantly more transfused than nonanemic patients (34.7% vs 5.6% *P* = .001), although once transfusion was indicated, the number of RBCs units transfused per patient was not different between anemic and nonanemic individuals (2.76 ± 0.42 vs 3.0 ± 0.53; *P* = .76).

These data corroborate the findings in the literature that preoperative anemia before orthopedic surgery greatly increases the risk of blood transfusion.^5^, ^8^

Jans et al reported in similar orthopedics surgical settings that 11% of the overall patients need transfusion with an important difference regarding to preoperative anemia. 31.6% of the patients with anemia prior to surgery were transfused compared with only 8.1% of previously nonanemic patients.^1^

In a large retrospective study with 2394 patients submitted to primary knee and hip arthroplasty, 23.7% of patients were anemic in the preoperative setting. The risk of blood transfusion was associated with the presence of anemia.^8^ This finding was confirmed in other reports where anemia was associated with an increased risk of blood transfusion.^9^

Blood transfusion is the treatment of choice for acute perioperative anemia. Restrictive trigger has been used by many centers to reduce the allogeneic perioperative transfusion for their patients.^10^, ^11^ However, even if restrictive trigger is used with less units transfused, blood transfusion is also associated with a worse postoperative outcome in surgical and critically ill patients. A large study that compared the effect of red cell transfusion showed higher rates of morbidity and mortality in patients receiving one unit of red cells compared with patients who were not transfused.^12^

Therefore, the application of restrictive transfusion criteria is not sufficient to improve the outcomes after surgeries, and additional strategies should be implemented. These include optimization of a patient's preoperative hemoglobin concentration and reduction of surgical and iatrogenic blood losses. All these measures ideally should be delivered within the context of a multidisciplinary and multimodal Patient Blood Management (PBM) program.

The majority of hip and knee surgeries are elective, and postponing surgery for optimization of preoperative anemia is almost always possible. Several studies have shown improved hemoglobin and decreased need for red cell transfusions with the treatment of preoperative anemia.^13^

In addition, it is important to highlight that the hazards of transfusion include immunomodulatory effects, risks of circulatory overload, transfusion reactions, and infectious complications, and blood is an increasingly scarce product dependent exclusively on voluntary donors and so must be used in a restrictive and rational way.

One question that is yet unclear is if anemia reflects the severity of an underlying disease or is casual in leading to poor outcomes. We were unable to explore this, given the lack of detailed information on preoperative clinical conditions and perioperative blood loss. This is a weakness of our study. Further data points that we did not include were laboratory information pertinent to the etiology of the anemia, such as mean corpuscular volume, vitamin levels, and iron studies.

Another important finding in our report is that preoperative anemia was associated with longer hospital stay and higher rate of intensive care unit admission. Prolonged hospital stay is associated with a higher morbidity, poorer outcomes, and higher costs. Lunn and Elwood already described the association between preoperative anemia and worse clinical outcome in 1970.^14^

A meta‐analysis including over 900 000 patients who underwent major surgical procedures (including many orthopedic surgeries) confirmed that preoperative anemia, even if mild, is an independent risk factor for poorer postoperative outcomes.^15^ An observational cohort study of 38 770 patients from 474 hospitals in 27 countries, including low‐, middle‐, and high‐income countries, showed that 11 675 (30.1%) had preoperative anemia. Patients from low‐ and middle‐income countries have high prevalence of anemia, and the anemic patients have an increased risk of complications and death.^16^ Abdullah et al showed that that every gram increase in preoperative Hb reduced the patient's length of stay in the hospital by 0.2 days and that transfusion of 1 unit of blood is associated with adjusted odds ratio (aOR) of prolonged hospital stay length of 2.12 (*P* = .006), while transfusion of 2 or more units has an aOR of 6.71 (*P* < .001).^8^

Our study adds to the growing body of publications about the negative impact of preoperative anemia on postoperative outcomes in orthopedic surgeries. We found prolonged hospital stays and higher rate of intensive care admission. Our study found no difference in mortality between anemic and nonanemic patients. International guidelines recommend early detection of preoperative anemia to identify the cause and treat any underling reversible etiology, such as iron deficiency. It has been demonstrated that the treatment of anemia can reduce postoperative blood transfusion, LOS, and 30‐day readmission. Previous studies have already reported that preoperative intravenous iron treatment of iron‐deficiency anemia in patients who underwent major abdominal surgery resulted in reduction of median hospital stay by 3 days.^17^ Similar results were achieved in the United Kingdom and Australia in elective hip/knee arthroplasty.^10^, ^18^

Although this information is already available, in many institutions, detection, evaluation, and management of anemia are left to the physician discretion. Jung‐Konig et al reported recently a survey performed in 10 European hospitals from seven different countries from the “Patient Blood Management in Europe Working Group.”^9^ Organization and management of preoperative anemia were heterogeneous in those hospitals. Almost all hospitals had pathways for managing preoperative anemia in place; however, only two nations had national guidelines. In six of the 10 participating hospitals, preoperative anemia management was organized by anesthetist, and the focus was on treating iron‐deficiency anemia. In less developed health systems, such as the Brazilian health system, few isolated initiatives can be identified.^11^ This study is part of a larger project to implement a PBM program in our institution.

## 6 CONCLUSIONS

In conclusion, our study demonstrated that preoperative anemia is prevalent (30.6%) in candidates for elective hip and knee arthroplasty, and preoperative anemia was associated with an increased risk of transfusion and prolonged LOS in men and women and higher rate of intensive care unit admission for female. Patient blood management programs aimed at optimizing preoperative hemoglobin level; minimizing the risk of bleeding and increasing anemia tolerance are necessary.

## FUNDING

This research received research grants from Rocha Brito Foundation. This grant supported statistical professionals for the analysis of the data.

## CONFLICT OF INTEREST

There was no conflict of interest.

## TRANSPARENCY STATEMENT

Gustavo de Carvalho Duarte affirms that this manuscript is an honest, accurate, and transparent account of the study being reported; that no important aspects of the study have been omitted; and that any discrepancies from the study as planned (and, if relevant, registered) have been explained. The authors confirm that the data supporting the findings of this study are available within the article.

## AUTHORS CONTRIBUTION

Conceptualization: Gustavo de Carvalho Duarte, José Francisco Comenalli Marques Júnior, Aguinaldo Pereira Catanoce, José Luiz Zabeu, and Nathália Aparecida Gonçalves de Oliveira.

Data Curation: Nathália Aparecida Gonçalves de Oliveira and Gustavo de Carvalho Duarte.

Formal Analysis: Glaciano Nogueira Ribeiro, Alfredo Mendrone‐Júnior, and Mariangela Moschen.

Writing—Original Draft: Gustavo de Carvalho Duarte and Alfredo Mendrone‐Júnior.

Writing—Review and Editing: Gustavo de Carvalho Duarte, Alfredo Mendrone‐Júnior, Glaciano Nogueira Ribeiro, and Dante Mario Langhi.

All authors have read and approved the final version of the manuscript.

Gustavo de Carvalho Duarte had full access to all of the data in this study and takes complete responsibility for the integrity of the data and the accuracy of the data analysis.
